# Beliefs and Communication Practices Regarding Cognitive Functioning Among Consumers and Primary Care Providers in the United States, 2009

**DOI:** 10.5888/pcd10.120249

**Published:** 2013-04-18

**Authors:** Daniela B. Friedman, India D. Rose, Lynda A. Anderson, Rebecca Hunter, Lucinda L. Bryant, Bei Wu, Angela J. Deokar, Winston Tseng

**Affiliations:** Author Affiliations: Daniela B. Friedman, University of South Carolina, Columbia, South Carolina; Lynda A. Anderson, Centers for Disease Control and Prevention and Emory University, Atlanta, Georgia; Rebecca Hunter, University of North Carolina at Chapel Hill, Chapel Hill, North Carolina; Lucinda L. Bryant, University of Colorado, Denver, Colorado; Bei Wu, Duke University, Durham, North Carolina; Angela J. Deokar, Centers for Disease Control and Prevention, Atlanta, Georgia; Winston Tseng, University of California, Berkeley, California.

## Abstract

**Introduction:**

Limited research has examined primary care providers’ communication with patients about maintaining cognitive functioning. Our study’s objective was to compare the perceptions of consumers and primary care providers related to beliefs and communication practices about lifestyle behaviors beneficial for overall health and for maintaining cognitive functioning.

**Methods:**

In 2009, we submitted 10 questions to Porter Novelli’s HealthStyles survey and 6 questions to their DocStyles survey. We compared consumers’ (n = 4,728) and providers’ (n = 1,250) beliefs, practices, and information sources related to maintaining health and cognitive functioning. We made comparisons using nonparametric statistics.

**Results:**

Approximately 76% of consumers considered their health to be good or very good; 73.4% were concerned or very concerned about the possibility that their memory may worsen with age. Women were significantly more concerned than men, and white consumers were more concerned than black and Hispanic consumers. Consumers reported they believed that intellectual stimulation (86.6%), physical activity (82.6%), and healthful diet (82.5%) prevented or delayed cognitive impairment. Providers reported advising patients to reduce cognitive impairment risk through physical activity (85.9%), intellectual stimulation (80.3%), and social involvement (67.4%). Few consumers (7.8%) reported receiving this information from providers but reported learning about strategies to maintain memory, primarily from television (50.1%), magazines (44.1%), and newspapers (33.7%).

**Conclusion:**

Providers reported advising patients about how to reduce risks of cognitive impairment. Consumers reported receiving this information from other sources. Findings suggest a need to examine and assess media messages and to better understand patient–provider communication about cognitive functioning.

## MEDSCAPE CME

Medscape, LLC is pleased to provide online continuing medical education (CME) for this journal article, allowing clinicians the opportunity to earn CME credit.

This activity has been planned and implemented in accordance with the Essential Areas and policies of the Accreditation Council for Continuing Medical Education through the joint sponsorship of Medscape, LLC and Preventing Chronic Disease. Medscape, LLC is accredited by the ACCME to provide continuing medical education for physicians.

Medscape, LLC designates this Journal-based CME activity for a maximum of 1 **AMA PRA Category 1 Credit(s)™**. Physicians should claim only the credit commensurate with the extent of their participation in the activity.

All other clinicians completing this activity will be issued a certificate of participation. To participate in this journal CME activity: (1) review the learning objectives and author disclosures; (2) study the education content; (3) take the post-test with a 70% minimum passing score and complete the evaluation at www.medscape.org/journal/pcd (4) view/print certificate.

**Release date: April 17, 2013; Expiration date: April 17, 2014**

### Learning Objectives

Upon completion of this activity, participants will be able to:

- Describe the perceptions of consumers related to beliefs and communication practices about lifestyle behaviors beneficial for overall health and for maintaining cognitive functioning
- Describe the perceptions of primary care providers related to beliefs and communication practices about lifestyle behaviors beneficial for overall health and for maintaining cognitive functioning
- Describe potential discrepancies in communications between patients and their providers about cognitive impairment and strategies to promote more effective communications

**EDITORS**

Rosemarie Perrin, Editor, Camille Martin, Editor; *Preventing Chronic Disease*. Disclosure: Disclosure: Rosemarie Perrin and Camille Martin have disclosed no relevant financial relationships.

**CME AUTHOR**

Laurie Barclay, MD, freelance writer and reviewer, Medscape, LLC. Disclosure: Laurie Barclay, MD, has disclosed no relevant financial relationships.

**AUTHORS AND CREDENTIALS**

Disclosures: Daniela Friedman, PhD; India Rose, PhD; Lynda Anderson, PhD; Rebecca Hunter, MEd; Lucinda L. Bryant, PhD; Bei Wu, PhD; Angela J. Deokar, MPH, CHES, CPH; and Winston Tseng, PhD have disclosed no relevant financial relationships.

Affiliations: Daniela Friedman, University of South Carolina, Columbia, South Carolina; Lynda Anderson, Centers for Disease Control and Prevention and Emory University, Atlanta, Georgia; Rebecca Hunter, University of North Carolina at Chapel Hill, Chapel Hill, North Carolina; Lucinda L. Bryant, University of Colorado, Denver, Colorado; Bei Wu, Duke University, Durham, North Carolina; Angela J. Deokar, Centers for Disease Control and Prevention, Atlanta, Georgia; Winston Tseng, University of California, Berkeley, California.

## Introduction

Alzheimer’s disease is the sixth leading cause of death among US adults (1). While death rates for other leading causes of death, such as cancer, are declining, rates for Alzheimer’s disease and other forms of dementia are on the rise. Experts report that between 2.6 and 5.1 million Americans may have Alzheimer’s disease (2), and the numbers are expected to more than double by 2050 (3) unless more effective ways to prevent and treat the disease are identified and implemented. Risk factors such as physical inactivity and poor diet, which are associated with such chronic conditions as heart disease and stroke, also are associated with cognitive decline in humans (4–8). There is no unequivocal scientific evidence demonstrating that altering diet and physical activity through interventions results in improved cognitive functioning (9). Moreover, the general public and people caring for people living with Alzheimer’s disease express fear of the disease (10–12). Popular media sometimes state that physical and mental exercises may promote cognition (13–17), but older adults report that media messages like these are often vague and conflicting (18).

Consumers in general consider their primary care providers to be their most trusted source of health information (19,20). An ASA-MetLife Foundation survey concerning cognitive functioning showed that physicians are consumers’ primary source for information (21). A recent study using national data from the 2008 DocStyles survey (22) reported that primary care physicians who discussed reducing risk of cognitive impairment with their patients were most likely to give advice about physical activity, intellectual stimulation, and healthful diet. More than 80% of physicians, however, indicated that strength of scientific evidence on risk reduction for cognitive impairment was moderate to very weak (23).

The primary objective of this study was to compare the perceptions of consumers and primary care providers related to beliefs and communication practices about lifestyle behaviors beneficial for overall health and for maintaining cognitive functioning. The secondary objective was to obtain information for developing future communications and education programs by examining consumers’ and providers’ perceptions on the basis of demographics and exploring the sources of information consumers and providers use.

## Methods

We conducted a descriptive study of Porter Novelli survey results (24–26) using nonparametric tests. Porter Novelli conducts annual surveys of diverse populations; surveys include a mail survey of the general public, HealthStyles (24,25), and a Web-based survey of providers, DocStyles (26–30). The Centers for Disease Control and Prevention (CDC) licenses the results of these 2 surveys from Porter Novelli. Although Porter Novelli and its vendors are not subject to CDC institutional review board review, they do adhere to all professional standards and codes of conduct set by the Council of American Survey Research Organizations (CASRO). Respondents are informed that their answers are being used for market research, that they may refuse to answer any question at any time, and that no personal identifiers are included in their data file.

We engaged a panel of 8 members from the CDC’s Healthy Aging Research Network to develop a set of questions to submit to the 2009 HealthStyles and DocStyles surveys (24–26). These questions addressed beliefs about cognitive functioning, risk reduction, and sources of information for consumers and primary care providers.

### Survey questions

We used data from the 2009 HealthStyles survey (24,25) to examine consumers’ beliefs about 1) the possibility that consumers’ memory may worsen as they age, 2) activities to stay healthy, 3) activities that may benefit consumers’ ability to think or remember, 4) sources of information from which consumers could learn ways to maintain their ability to think and remember things, and 5) whether providers recently talked with consumers about ways to stay mentally “sharp.” Using data from the 2009 DocStyles survey (26), we examined primary care providers’ beliefs about 1) advice given to patients about maintaining and promoting their health, 2) advice given to patients about preventing or delaying cognitive impairment and dementia, 3) the benefits of an early diagnosis of cognitive impairment, 4) talking to their patients about cognition, and 5) sources of information used to obtain information about new evidence and practice guidelines related to cognitive impairment and dementia. Respondents to both surveys (consumers and providers) selected responses that applied to them (Appendix).

### Survey sampling

The 2009 consumer data were collected in waves by Synovate, Inc, via their existing consumer mail panel. Synovate administered the initial wave, called ConsumerStyles, from April through May 2009. Stratified random sampling was used to generate a list of 21,420 potential respondents aged 18 years or older who received the ConsumerStyles survey, of whom 10,587 responded. In September and October 2009, Synovate sent HealthStyles version B (final wave of 2009 surveys) to 6,504 respondents who had completed the ConsumerStyles survey earlier in the year. Responses to HealthStyles, including cognitive functioning questions, were received from 4,728 consumer respondents, yielding a response rate of 72.7%. Respondents were not required to participate and no personal identifiers were included in the data set.

In our analysis, we weighted the HealthStyles data by sex, age, income, race, and household size to reflect the distribution of the 2008 US Current Population Survey (31). Assumed homogeneity of views shared by subgroups of the population provides the basis for weighting these variables. We analyzed data using SPSS version 19.0 (IBM Statistics for Windows, Version 19.0. Armonk, New York). Descriptive statistics for both consumers and providers included respondents’ demographics (age, race, sex) and sources of cognitive functioning information. For consumers, statistics included beliefs about healthy lifestyle and cognition; for providers, statistics included years in practice, practice setting, and types of advice for healthy lifestyle and cognition. Responses from primary care physicians and nurse practitioners were combined if differences between findings for these 2 provider groups were not significant. To develop meaningful categories that captured trends in the data, we categorized the age of health care providers and consumers as 34 years or younger, 35 to 55 years, and 56 years or older. We conducted multiple comparisons with χ^2^ tests with significance levels set at *P* ≤ .01 to contrast differences between health care providers’ and consumers’ perceptions about lifestyle factors and reduction of risk factors.

In July 2009, the DocStyles survey was administered to a main sample of primary care physicians (family and general-practice physicians, internists) and additional samples of other provider types, including pediatricians, dermatologists, nurse practitioners, and registered dietitians. The sample of physicians was drawn from the Epocrates Honors Panel, an opt-in panel of more than 156,000 physicians verified by using the American Medical Association’s (AMA’s) master file. Epocrates randomly selected eligible providers to match the AMA master file proportions for age, sex, and region. The nurse practitioner sample was drawn from Epocrates’ Allied Health Panel consisting of 473,000 health professionals, including 44,523 nurse practitioners. We selected respondents who practice in the United States; actively see patients; work in an individual, group, or hospital practice; and have been in practice for at least 3 years.

Only primary care physicians and nurse practitioners were asked the questions about cognitive functioning and impairment. Porter Novelli set response quotas to reach 1,000 primary care physicians and 250 nurse practitioners. To reach the 1,000 quota for primary care physicians, Porter Novelli recruited 2,325 primary care physicians; 1,000 completed the survey, 43 had incomplete surveys, 52 were eliminated by screener questions, and 1,230 did not respond to the invitation or responded after the survey closed, resulting in a 45.0% formula-adjusted response rate. We excluded those who did not complete the survey (n = 43) or were eliminated because of ineligibility (n = 52) (22,23,26). To reach the quota for nurse practitioners, Porter Novelli recruited 500 nurse practitioners; 250 completed the survey, 13 had incomplete surveys, 11 were eliminated by screener questions, and 226 did not respond to the invitation or responded after the survey closed, resulting in a 52.0% formula-adjusted response rate. Providers’ participation was optional and no personal identifiers were included in the data set.

## Results

More than half of consumers were women, with an average age of 46.4 years (range, 18–99 y). More than two-thirds reported their race as white, and 54.9% were married (Table 1). Over three-quarters of the sample (76.3%) rated their overall general health as very good or good.

**Table 1:** Demographic Characteristics of Survey Respondents, HealthStyles (24,25) and DocStyles (26), 2009^a^

Characteristic	Consumers^b^ (n = 4,728)	Physicians (General/Family Physicians, Internists) (n = 1,000)	Nurse Practitioners (n = 250)
**Average years in practice (n)**	**—** c	**14.7**	**16.4**
**Sex**			
Male	2,293 (48.5)	718 (71.8)	32 (12.8)
Female	2,435 (51.5)	282 (28.2)	218 (87.2)
**Age, y**			
≤34	1,442 (30.5)	113 (11.3)	30 (12.0)
35–55	1,810 (38.3)	702 (70.2)	170 (68.0)
≥56	1,476 (31.2)	185 (18.5)	50 (20.0)
**Race/ethnicity**			
White	3,257 (68.9)	722 (72.2)	236 (94.4)
Black	543 (11.5)	46 (4.6)	4 (1.6)
Hispanic	633 (13.4)	45 (4.5)	3(1.2)
Other	295 (6.2)	187 (18.7)	7 (2.8)
**Education**			
Not high school graduate	257 (5.4)	—c	—c
High school graduate	1,163 (24.6)	—c	—c
Attended college	1,791 (37.9)	—c	—c
College degree	1,517 (32.1)	—c	—c
**Marital status**			
Married	2,595 (54.9)	—c	—c
Not married^d^	2,133 (45.1)	—c	—c

Abbreviation: CI, confidence interval.

a Pairwise χ^2^ tests with Bonferonni correction were used to compare variables with significant differences; all data are presented as n (%) unless otherwise indicated.

b Consumer data from the HealthStyles (24,25) survey were weighted by sex, age, income, race, and household size.

c Indicates response option was not included on the survey.

d Includes single, divorced, and widowed.

Most physician respondents were men, with an average age of 45 years (range, 27–95 y). On average, they had practiced medicine for 14.7 years (range, 3–40 y). Approximately 61% reported being family or general physicians; 39% were internists. Nurse practitioners were primarily women, with an average age of 46.1 years (range, 27–67 y). On average, they had practiced for 16.4 years (range, 3–46 y) (Table 1).

Among consumers, 73.4% indicated being “somewhat” or “very much” concerned about the possibility that their memory may worsen as they age. Consumers who reported fair or poor overall health (n = 663, 14.0%) were more likely to be very much concerned about their memory (fair, 34.3%; poor, 44.2%) than those who reported excellent health (17.0%), very good health (14.6%), or good health (21.5%) (*P* < .001). Women reported significantly greater levels of concern about their memory than men (58.0% vs 42.0%; *P* < .001). Whites were significantly more likely to report being very much concerned (64.0%) than black (14.6%) or Hispanic (14.8%) respondents (*P* < .001). Consumers aged 35 to 55 years (43.3%) were significantly more likely to be very much concerned about their memory worsening compared with those aged 34 or younger (24.5%) and those aged 56 or older (32.1%) (*P* < .001) (data not shown).

### Consumer beliefs vs provider advice about ways to maintain a healthy lifestyle

Consumers most frequently reported that they engaged in the following health behaviors: obtaining intellectual stimulation (74.0%), being physically active (71.9%), eating a healthful diet (65.4%), taking vitamins or dietary supplements (60.6%), avoiding smoking (59.0%), maintaining a healthy weight (55.4%), engaging in social activity (51.8%), and taking prescribed medications (37.6%) (Table 2). Primary care providers (primary care physicians and nurse practitioners combined) most frequently reported giving advice to patients about maintaining and promoting health by engaging in physical activity (98.2%), maintaining a healthy weight (91.9%), eating a healthful diet (91.8%), and limiting alcohol consumption (75.3%).

**Table 2:** Consumers’ Perceptions and Providers’ Self-Reported Advice About Maintaining a Healthy Lifestyle and Reducing Cognitive Impairment, HealthStyles (24,25) and DocStyles (26), 2009^a^

Providers’ Advice and Consumers’ Perceptions	How to Maintain and Promote Own Health, n (%)^b^			How to Prevent or Delay Cognitive Impairment, n (%)^b^		

	Consumers (n = 4,728)^c^	Physicians (n = 1,000)	Nurse Practitioners (n = 250)	Consumers (n = 4,728)^c^	Physicians (n = 1,000)	Nurse Practitioners (n = 250)
Be physically active	3,399 (71.9)	981 (98.1)	247 (98.8)	3,905 (82.6)	861 (86.1)	213 (85.2)
Maintain a healthy weight	2,619 (55.4)	915 (91.5)	234 (93.6)	3,059 (64.7)	457 (45.7)	107 (42.8)
Eat a healthful diet	3,092 (65.4)	914 (91.4)	233 (93.2)	3,900 (82.5)	609 (60.9)	163 (65.2)
Limit alcohol	—d	748 (74.8)	193 (77.2)	—d	591 (59.1)	143 (57.2)
Be socially active	2,449 (51.8)	481 (48.1)	121 (48.4)	3,030 (64.1)	667 (66.7)	175 (70.0)
Get intellectual stimulation	3,498 (74.0)	413 (41.3)	100 (40.0)	4,094 (86.6)	802 (80.2)	202 (80.8)
Avoid polypharmacy	—d	386 (38.6)	90 (36.0)	—d	411 (41.1)	105 (42.0)
Take vitamins/supplements	2,865 (60.6)	306 (30.6)	109 (43.6)	3,030 (64.1)	293 (29.3)	91 (36.4)
Avoid smoking	2,790 (59.0)	—d	—d	2,449 (51.8)	—d	—d
Take prescribed medication	1,777 (37.6)	160 (16.0)	35 (14.0)	1,485 (31.4)	116 (11.6)	25 (10.0)
None of these	85 (1.8)	20 (0.2)	3 (0.1)	99 (2.1)	40 (4.0)	20 (8.0)

Abbreviation: CI, confidence interval.

a Pairwise χ^2^ tests with Bonferonni correction were used to compare variables with significant differences.

b Percentages sum to more than 100% because respondents were asked to select all that apply.

c Consumer data from the HealthStyles survey were weighted by sex, age, income, race, and household size.

d Indicates response option was not included on the survey.

### Consumer beliefs versus provider advice about reducing risk of cognitive impairment

Most consumers reported that becoming or staying mentally stimulated (86.6%), being physically active (82.6%), and eating a healthful diet (82.5%) could prevent or delay cognitive impairment (Table 2). They also indicated that maintaining a healthy weight (64.7%), being socially involved (64.1%), taking vitamins or dietary supplements (64.1%), avoiding smoking (51.8%), and taking prescribed medication (31.4%) could prevent or delay cognitive impairment. In response to the question about whether a provider talked with them in the past 12 months about ways to stay mentally sharp, only 7.8% of consumers responded yes.

To prevent or delay cognitive impairment and dementia among their patients, primary care providers most often advised patients to engage in physical activity (85.9%), obtain intellectual stimulation (80.3%), be socially active (67.4%), eat a healthful diet (61.8%), limit alcohol consumption (58.7%), and maintain a healthy weight (45.1%). Number of years in practice was not significantly related to differences in advice given.

Nearly 72% of providers agreed with the statement that an early diagnosis of cognitive impairment, dementia, or Alzheimer’s disease was beneficial because it provides opportunities to patients and their families for treatment and planning. Approximately 15.8% of providers reported feeling neutral about the benefits of an early diagnosis; 9.7% disagreed or strongly disagreed that there were benefits to early diagnosis; 2.9% said benefits could not be determined from the current scientific evidence.

### Information sources about cognitive functioning

Consumers obtained most of their cognition information from public media including television (50.1%), followed by magazines (44.1%), newspapers (33.7%), Internet or professional websites (27.2%), and radio (17.5%). Primary care providers reported that they obtained information about new evidence and practice guidelines related to cognitive functioning from professional journals (43.8%), followed by professional websites or listservs (19.4%), online and in-person continuing medical education (11.4%), scientific meetings or conferences (10.6%), and through personal digital assistant or BlackBerry (BlackBerry, Waterloo, Ontario, Canada) (4.2%). Very few providers obtained cognition information from popular media (0.2% each for newspaper, television, and radio).

## Discussion

To our knowledge, ours is the first study to examine and compare the beliefs and communication practices of consumers and primary care providers regarding cognitive functioning. Consumers and providers agreed that physical activity and mental stimulation are factors in maintaining cognitive functioning. Providers most often reported that they advise patients to engage in physical activity and intellectual stimulation to prevent or delay impairments in cognitive functioning. Providers were less likely than consumers to believe that intellectual stimulation was important in maintaining and promoting overall health. Consumers also more strongly considered vitamins and supplements to be beneficial to general health and cognitive functioning. Consumer perception regarding the importance of intellectual stimulation is consistent with previous qualitative research conducted with ethnically diverse older consumers where participants reported that mental stimulation, particularly reading, and mental exercises (eg, puzzles, games) promoted cognitive functioning (32). That same research found that older consumers reported confusion regarding the role of dietary supplements from media messages. This is interesting given that 64% of consumers who responded to the 2009 HealthStyles survey believe they can prevent or delay cognitive impairment with vitamins and dietary supplements. Other research indicates that understanding and improving consumers’ main sources of health and cognition information (eg, general mass media) will be critical for the development of effective public health interventions on cognitive impairment and dementia (13–16).

Our results support prior findings that consumers are concerned about cognitive impairment (10–12). Women, whites, and people aged 35 to 55 were significantly more likely to be concerned than men, other racial/ethnic groups, and people in the other age categories, respectively. Findings from other research (18,32) suggest that concern may arise from confusion about current cognitive impairment messages from various sources, including mass media. Communication efforts aimed at reaching consumers could potentially benefit from focusing on messages designed to address possible misperceptions and misinformation regarding cognitive impairment. Primary care providers can play a key role in communication about this issue, but our findings indicate a discrepancy between consumers’ and providers’ reports of discussions about cognitive functioning. Because most providers agreed that early diagnosis of Alzheimer’s disease and other dementias is beneficial, it seems important to reduce possible communication issues.

This study had limitations. First, data from HealthStyles and DocStyles surveys are cross-sectional, and causality cannot be inferred. Second, the information is self-reported; consumers’ and providers’ actual perceptions and behaviors may differ from self-reports. Third, we gave respondents only structured responses for each question; the available options may have influenced their responses. Fourth, the different perspectives of the 2 surveys — consumers were responding to a consumer health survey and providers were describing their practices and beliefs with respect to their patients — restricted our ability to make direct comparisons between consumers and providers. Fifth, the DocStyles survey data were not weighted to reflect a general population of providers. Primary care providers were randomly selected from Epocrates panels that may not accurately represent all US physicians and nurse practitioners. Sixth, although the HealthStyles survey data were weighted, the assumption that population subgroups have similar views, upon which the weighting is based, may not be accurate. Despite these limitations, this large sample of US consumers and primary care providers provides evidence about their perceptions and self-reported practices for reducing cognitive impairment or dementia risk.

Our findings have implications for future research. Results suggest that the general public may not be obtaining the full spectrum of information about cognition from primary care providers, or perhaps not recalling the information. At the same time, the providers in this study report giving advice to their patients regarding how to reduce the risk of cognitive impairment. Thus, communications research is needed to explore this discrepancy and to determine how best to enhance patient–provider communications about cognitive functioning, especially among groups that are most concerned about future memory losses and dementia (eg, women, middle-aged consumers). Identifying effective strategies for discussions about cognitive functioning while we continue to build the evidence base for cognition recommendations should improve patient–provider communication. Future research also should examine how providers determine whether a patient has a cognitive impairment, what, if any, screening or assessment tools are used, and how this information is communicated to patients, family, and caregivers. Our findings indicate a need to understand potential discrepancies in communications between patients and their providers about cognitive impairment and strategies to promote more effective communications.

Our findings also have implications and suggestions for the new topic area in Healthy People 2020, “Dementias, including Alzheimer’s disease” (33), and underscore the importance of increasing awareness about these conditions. With mortality rates for Alzheimer’s disease and other forms of dementia on the rise, our findings demonstrate the need to study the actual communications between consumers and their providers regarding cognitive impairment and to support the goals and strategies of the National Plan to Address Alzheimer’s Disease released in May 2012 (34). These findings are relevant to the National Plan’s goals of decreasing misperceptions about Alzheimer’s disease and related dementias through enhanced public awareness and engagement and connecting consumers, patients, and caregivers to accurate information and resources as well as enhancing quality and efficiency of care through provider education. We hope that our work can help promote awareness about this public health issue.

## Figures and Tables

**Table:** 

**1. The activity supported the learning objectives.**				
**Strongly Disagree**				**Strongly Agree**
1	2	3	4	5
**2. The material was organized clearly for learning to occur.**				
**Strongly Disagree**				**Strongly Agree**
1	2	3	4	5
**3. The content learned from this activity will impact my practice.**				
**Strongly Disagree**				**Strongly Agree**
1	2	3	4	5
**4. The activity was presented objectively and free of commercial bias.**				
**Strongly Disagree**				**Strongly Agree**
1	2	3	4	5

## Appendix. Research Questions and Response Options, HealthStyles (24,25) and DocStyles (26), 2009**^a^**

### Consumers (24,25)

**Concerns about cognitive impairment or decline**

*How concerned are you about the possibility your memory might get worse as you get older?*

- Not at all
- Very little
- Somewhat
- Very much
- Not specified

**Beliefs about ways to maintain a healthy lifestyle**

*What do you do to keep yourself healthy?*

- Be physically active
- Eat a healthy diet
- Take vitamins/minerals
- Be socially involved
- Avoid smoking
- Take medication
- Get or stay at a healthy weight
- Become or stay mentally stimulated
- Do not do any of these
- Other

**Beliefs about ways to reduce risk of cognitive impairment**

*What types of activities may benefit your ability to think or remember?*

- Be physically active
- Eat a healthy diet
- Take vitamins/minerals
- Be socially involved
- Avoid smoking
- Take medication
- Get or stay at a healthy weight
- Become or stay mentally stimulated
- Do not do any of these
- Other

**Sources of information about cognition**

*Where have you heard about ways to maintain your ability to think or remember things?*

- Newspaper
- Magazine
- Television
- Radio
- Internet
- Health care provider
- Friends or family
- None of these
- Not specified

**Communication with health care provider**

*In the past 12 months, has a health care professional talked to you about ways to maintain your ability to think or remember things (ways to stay mentally sharp)?*

- Yes
- No

### Health Care Providers (26)

**Reported advice about healthy lifestyle**

*Specifically, what types of advice do you give your adult patients about maintaining and promoting their health?*

- Be physically active
- Attain or maintain a healthy weight
- Take nutritional supplements
- Get intellectual stimulation
- Take certain medications
- Eat a healthful diet
- Be socially active
- Limit consumption of alcohol
- Avoid polypharmacy
- None of these

**Reported advice about reducing risk of cognitive impairment**

*Specifically, what types of advice do you give your adult patients about preventing or delaying cognitive impairment and dementia?*

- Be physically active
- Attain or maintain a healthy weight
- Take nutritional supplements
- Get intellectual stimulation
- Take certain medications
- Eat a healthful diet
- Be socially active
- Limit consumption of alcohol
- Avoid polypharmacy
- None of these

**Benefits of an early diagnosis**

*An early diagnosis of cognitive impairment, dementia, or Alzheimer’s disease is beneficial because it provides opportunities for treatment and planning to patients and their family.*

- Strongly agree
- Agree
- Neutral
- Disagree
- Strongly disagree
- Cannot be determined from current scientific evidence

**Sources of information about cognition**

*What is the major source that you use to obtain information about new evidence and practice guidelines related to cognitive impairment and dementia?*

- Professional journals
- Professional websites/listservs
- Brochure/booklet
- Scientific meeting/conference
- PDA or BlackBerry
- Popular media (eg, newspapers, radio, television, magazines)
- Online/in-person continuing medical education
- Drug or pharmaceutical representative
- None of these sources
- Not applicable to practice

^a^ We developed and submitted 10 questions for the 2009 HealthStyles survey and 6 questions for the 2009 DocStyles survey. This article describes findings from 5 HealthStyles questions and 4 DocStyles questions.

## Post-Test Information

To obtain credit, you should first read the journal article. After reading the article, you should be able to answer the following, related, multiple-choice questions. To complete the questions (with a minimum 70% passing score) and earn continuing medical education (CME) credit, please go to http://www.medscape.org/journal/pcd. Credit cannot be obtained for tests completed on paper, although you may use the worksheet below to keep a record of your answers. You must be a registered user on Medscape.org. If you are not registered on Medscape.org, please click on the "Register" link on the right hand side of the website to register. Only one answer is correct for each question. Once you successfully answer all post-test questions you will be able to view and/or print your certificate. For questions regarding the content of this activity, contact the accredited provider, CME@medscape.net. For technical assistance, contact CME@webmd.net. American Medical Association's Physician's Recognition Award (AMA PRA) credits are accepted in the US as evidence of participation in CME activities. For further information on this award, please refer to http://www.ama-assn.org/ama/pub/about-ama/awards/ama-physicians-recognition-award.page. The AMA has determined that physicians not licensed in the US who participate in this CME activity are eligible for **AMA PRA Category 1 Credits™**. Through agreements that the AMA has made with agencies in some countries, AMA PRA credit may be acceptable as evidence of participation in CME activities. If you are not licensed in the US, please complete the questions online, print the AMA PRA CME credit certificate and present it to your national medical association for review.

## Post-Test Questions

### Article Title: Beliefs and Communication Practices Regarding Cognitive Functioning Among Consumers and Primary Care Providers in the United States, 2009

**CME Questions**

1. You are consulting with a primary care clinic regarding strategies to maintain patients' overall health and cognitive functioning. Based on the study by Dr Friedman and colleagues, which of the following statements about the perceptions of consumers related to beliefs and communication practices about lifestyle behaviors beneficial for these goals is **most likely** correct?
  - Less than half of consumers considered their health to be good or very good
  - Men, Hispanics, and people over 60 years of age were most likely to be concerned or very concerned about the possibility that their memory may worsen with age
  - More than 75% of consumers thought that strategies to prevent or delay cognitive impairment were intellectual stimulation, physical activity, and healthful diet
  - Overall, less than half of consumers were concerned or very concerned about the possibility that their memory may worsen with age
2. Based on the study by Dr Friedman and colleagues, which of the following statements about the perceptions of primary care providers related to beliefs and communication practices regarding lifestyle behaviors beneficial for overall health and for maintaining cognitive functioning is **most likely** to appear in your report?
  - About half of providers reported advising patients to reduce cognitive impairment risk through physical activity, intellectual stimulation, and social involvement
  - About half of primary care providers reported advising patients to promote health through physical activity, maintaining a healthy weight, healthful diet, and limiting alcohol intake
  - Less than half of providers agreed that early diagnosis of cognitive impairment, dementia, or Alzheimer's disease was beneficial
  - Number of years in practice was not significantly related to differences in advice given
3. Based on the study by Dr. Friedman and colleagues, which of the following statements about potential discrepancies in communications between patients and their providers about cognitive impairment and strategies to promote more effective communications would **most likely** be correct?
  - About 75% of consumers reported receiving information from their providers about strategies to maintain memory
  - Providers were more likely than consumers to believe that intellectual stimulation was important in maintaining and promoting overall health.
  - Providers were more likely than consumers to strongly consider vitamins and supplements to benefit general health and cognitive functioning
  - The findings suggest a need to examine and assess media messages and to better understand patient–provider communication about cognitive functioning

**Evaluation**
